# Mitophagy Receptors in Tumor Biology

**DOI:** 10.3389/fcell.2020.594203

**Published:** 2020-11-11

**Authors:** Yangchun Xie, Jiao Liu, Rui Kang, Daolin Tang

**Affiliations:** ^1^Department of Oncology, The Second Xiangya Hospital, Central South University, Changsha, China; ^2^The Third Affiliated Hospital, Guangzhou Medical University, Guangzhou, China; ^3^Department of Surgery, University of Texas Southwestern Medical Center, Dallas, TX, United States

**Keywords:** mitophagy, cancer, cell death, autophagy, mitochondria

## Abstract

Mitochondria are multifunctional organelles that regulate cancer biology by synthesizing macromolecules, producing energy, and regulating cell death. The understanding of mitochondrial morphology, function, biogenesis, fission and fusion kinetics, and degradation is important for the development of new anticancer strategies. Mitophagy is a type of selective autophagy that can degrade damaged mitochondria under various environmental stresses, especially oxidative damage and hypoxia. The key regulator of mitophagy is the autophagy receptor, which recognizes damaged mitochondria and allows them to enter autophagosomes by binding to MAP1LC3 or GABARAP, and then undergo lysosomal-dependent degradation. Many components of mitochondria, including mitochondrial membrane proteins (e.g., PINK1, BNIP3L, BNIP3, FUNDC1, NIPSNAP1, NIPSNAP2, BCL2L13, PHB2, and FKBP8) and lipids (e.g., cardiolipin and ceramides), act as mitophagy receptors in a context-dependent manner. Dysfunctional mitophagy not only inhibits, but also promotes, tumorigenesis. Similarly, mitophagy plays a dual role in chemotherapy, radiotherapy, and immunotherapy. In this review, we summarize the latest advances in the mechanisms of mitophagy and highlight the pathological role of mitophagy receptors in tumorigenesis and treatment.

## Introduction

**FIGURE 1:**
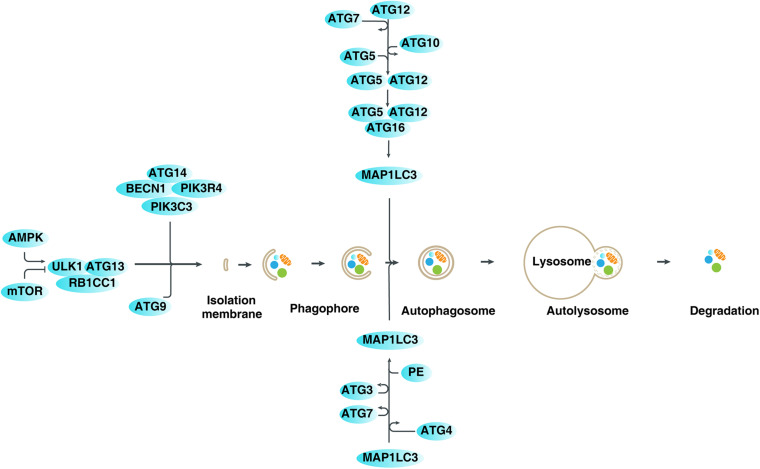
Mechanisms and regulation of autophagy in mammalian cells. Autophagy is a dynamic process involving the formation of several specific membrane structures, such as phagophores, autophagosomes, and autolysosomes. ATG proteins, in association with various regulators, are involved in regulating the dynamic process of membrane structure formation, leading to the degradation of various cargoes in lysosomes.

Autophagy, which was first observed under an electron microscope by Belgian scientist Christian de Duve in the 1950s, is a cellular phenomenon of “self-eating” by lysosomes (Yang and Klionsky, 2010). At present, based on the transport mode of cytosolic cargoes to lysosomes, autophagy is mainly divided into three types: macroautophagy (hereafter referred to as autophagy), microautophagy, and chaperone-mediated autophagy (Dikic and Elazar, 2018). As an important degradation mechanism, the process of autophagy involves the formation of lipid-related autophagosomes by wrapping various cargoes (e.g., damaged organelles, unused proteins, and invading pathogens), and then fusing them with lysosomes to form autophagosomes and degrading their contents (Klionsky and Emr, 2000; Xie et al., 2020b). At the molecular level, autophagy-related (ATG) genes and proteins play a vital role in regulating the dynamic formation of autophagic membrane structures, mainly through protein-protein interactions (Kang et al., 2011; Dikic and Elazar, 2018; Figure 1). These ATG protein interactions are further modulated by various factors, especially kinase-mediated protein posttranslational modification (McEwan and Dikic, 2011; Xie et al., 2015). Generally, the activation of autophagy is an important defense mechanism that promotes cell survival and recovery under harmful stresses, such as starvation and hypoxia (Kroemer et al., 2010). The autophagic degradation products can be reused for protein synthesis and energy production, although the underlying mechanism of this process is unclear. In contrast, an excessive activation of autophagy may lead to cell death, which is called autophagy-dependent cell death (Bialik et al., 2018; Galluzzi et al., 2018; Tang et al., 2019). In particular, recent studies indicate that ferroptosis is a type of autophagy-dependent cell death (Hou et al., 2016; Bai et al., 2019; Li et al., 2020; Xie et al., 2020a), highlighting the importance of autophagy in the degradation of proteins involved in iron and lipid metabolism (Zhou et al., 2020; Liu et al., 2020). It is also worth noting that the term “autophagic cell death” is used to describe the phenotype of increased autophagy during the induction of cell death, regardless of the effect of autophagic response on cell fate (Kroemer and Levine, 2008). Therefore, autophagy plays a dual role in cell survival and cell death, which is related to human disease, especially cancer and neurodegenerative diseases (Levine and Kroemer, 2019).

Depending on whether specific autophagic receptors (also known as autophagic adaptor proteins) are needed to degrade specific substrates, autophagy can be non-selective or selective (Zaffagnini and Martens, 2016). In recent years, a large number of types of selective autophagy have been found to regulate cell homeostasis, such as mitophagy (Harper et al., 2018), pexophagy (Cho et al., 2018), lipophagy (Kounakis et al., 2019), ferritinophagy (Mancias et al., 2014), and clockophagy (Yang et al., 2019). Among them, mitophagy is the most-studied selective autophagy, which eliminates damaged or aging mitochondria by recognizing different components of mitochondrial structure via various autophagy receptors (Lemasters, 2005). Dysregulated mitophagy is closely related to many physiological and pathological processes, such as aging, neurodegenerative diseases, and cancer (Palikaras et al., 2018). In this review, we first introduce the structure and function of mitochondria, and then focus on the molecular mechanisms of mitophagy. Finally, we describe the pathologic role of mitophagy regulators in tumor development and therapy, and will discuss new directions for cancer treatment.

### Structure and Function of Mitochondria

Mitochondria are double-membrane organelles present in most eukaryotic cells, and their size, number, and appearance are different on different cells (Herst et al., 2017; Pfanner et al., 2019). Like chloroplasts in plants and algae, mitochondria may have evolved from primitive bacteria (Gray, 2012). The main chemical components of mitochondria include water, protein, and lipids. In addition, mitochondria have a small amount of small molecules, such as coenzymes and nucleic acids. Proteins, including soluble and insoluble proteins, account for 65 to 70% of the dry weight of mitochondria. Soluble proteins are mainly the enzymes located in the mitochondrial matrix and the periphery of the membrane, whereas insoluble proteins constitute the main body of the membrane, part of which is composed of mosaic proteins or enzymes (Pfanner et al., 2019). Lipids in mitochondria are mainly distributed in two layers of membranes, accounting for 20 to 30% of the dry weight. Phospholipids in mitochondria account for more than 75% of total lipids. The amount of phospholipids in the mitochondrial membrane of different tissues of the same organism is relatively stable. Abundant cardiolipin and less cholesterol are the obvious differences between the structure of mitochondria and other cell membranes (Montero et al., 2010).

From the outside to the inside, the mitochondria can be divided into four functional areas: the outer mitochondrial membrane (OMM), the intermembrane space (the space between the outer and inner membranes), the inner mitochondrial membrane (IMM), and the matrix (space within the inner membrane) (Pfanner et al., 2019). The OMM is smoother and acts as the boundary membrane of organelles, while the IMM folds inward to form mitochondrial cristae (e.g., lamellar cristae, tubular cristae, and vesicular cristae), which complicate biochemical reactions. Mitochondria are the main sites for oxidative phosphorylation and synthesis of adenosine triphosphate (ATP) in cells, and provide chemical energy for cellular activities as the “powerhouse of the cell.” In addition to supplying energy for cells, mitochondria are also involved in various processes, such as cell differentiation, signal transduction, cell growth, the cell cycle, and cell death (Herst et al., 2017; Bock and Tait, 2020). Dysfunctional mitochondria are unable to execute oxidative phosphorylation and consequently accumulate reactive oxygen species (ROS) in the cells. Mitochondrial oxidative stress is associated with a variety of pathologies, especially age-related diseases (e.g., cancer). In order to avoid mitochondrial dysfunction, some conservative mechanisms have evolved to control the quality of mitochondria. Among them, mitophagy plays a central role in preventing mitochondrial damage by promoting mitochondrial turnover. Understanding the signal transduction and molecular modification of mitophagy is important for improving the homeostasis of mitochondria (Palikaras et al., 2018).

### Molecular Mechanisms of Mitophagy

**FIGURE 2:**
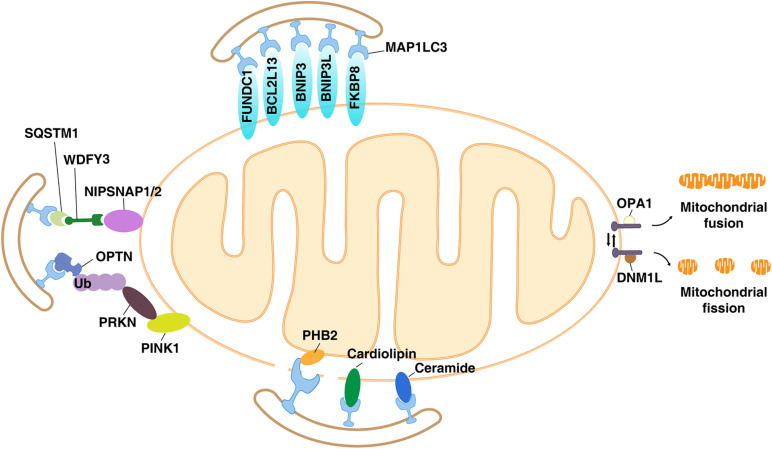
The Ub-dependent and Ub-independent receptor pathways of mitophagy. Many components of the mitochondria, including mitochondrial membrane proteins (e.g., PINK1, BNIP3L, BNIP3, FUNDC1, NIPSNAP1, NIPSNAP2, BCL2L13, PHB2, and FKBP8) and lipids (e.g., cardiolipin and ceramides), act as mitophagy receptors in a context-dependent manner.

Mitochondrial depolarization refers to the process in which the membrane potential of the mitochondria changes from negative to positive in the direction of depolarization (Zorova et al., 2018). During the electron transport process, as electrons flow down the chain of the redox complex located in the IMM, protons flow into the space between the IMM and the OMM. Therefore, the intermembrane space becomes positive, and the IMM becomes electrochemically polarized. The backflow of protons is related to the production of ATP. In this state, the mitochondria are polarized. When proton flow is independent of ATP production, mitochondria are considered to be depolarized (Zorova et al., 2018). Fission-induced mitochondrial depolarization is an important factor that triggers mitophagy to reduce oxidative stress (Twig and Shirihai, 2011). The molecular mechanisms involved in mitophagy are complex, and recognition of depolarized mitochondria requires a variety of cargo receptors and regulators. In general, mitophagy can be mediated through ubiquitin (Ub)-dependent and Ub-independent receptor pathways, as described below (Harper et al., 2018; Figure 2).

### Ub-Dependent Receptors

PTEN-induced kinase 1 (PINK1) is a serine/threonine protein kinase that localizes to mitochondria (Valente et al., 2004). Parkin RBR E3 ubiquitin protein ligase (PRKN/PARKIN/PARK2) is a component of the multiprotein E3 ubiquitin ligase complex, which can catalyze the covalent attachment of the ubiquitin part to the substrate protein (Shimura et al., 2000). Mutations in PINK1 and PRKN are implicated in Parkinson’s disease, an aging-related disease associated with mitochondrial abnormalities and motor nerve damage (Pickrell and Youle, 2015). Importantly, the activation of the PINK1-PRKN pathway is the first and most studied regulatory mechanism of mitophagy. When mitochondria are damaged or depolarized, PINK1 stabilizes on the OMM, where it recruits and activates PRKN (Vives-Bauza et al., 2010). After being transported from the cytoplasm to the mitochondria, PRKN ubiquitinates many OMM proteins [e.g., translocase of outer mitochondrial membrane 20 (TOMM20/TOM20), mitofusin 1 (MFN1), and mitofusin 2 (MFN2)], resulting in the recruitment of autophagy receptors such as sequestosome 1 (SQSTM1/p62) and optineurin (OPTN) (Geisler et al., 2010; Wong and Holzbaur, 2014). The ubiquitinated proteins with autophagy receptors are then bound to the autophagosome-associated proteins [microtubule-associated protein 1 light chain 3 (MAP1LC3/LC3/Atg8) or GABA type A receptor-associated protein (GABARAP)] via their LC3 interacting domain (LIR) to direct the isolation membrane/phagophore of growing autophagosomes to surround damaged mitochondria (Wild et al., 2014). Finally, engulfed mitochondria are degraded and eliminated in autolysosomes (Geisler et al., 2010; Wong and Holzbaur, 2014). The activity and function of PINK1 or PRKN in mitophagy is further regulated by various binding partners and changes in mitochondrial dynamics (e.g., mitochondrial fission). Of note, some mammalian cells (Eiyama and Okamoto, 2015) do not express PINK1 or PRKN, indicating that PINK1- and PRKN-mediated mitophagy may have tissue and cell-specific effects in preventing mitochondrial dysfunction.

### Ub-Independent Receptors

#### BCL2 Interacting Protein 3-Like

The BCL2 apoptosis regulator (BCL2) family includes pro-apoptotic proteins [e.g., BCL2-associated X, apoptosis regulator (BAX), BCL2 antagonist/killer 1 (BAK1/BAK), and BH3 interacting domain death agonist (BID)] and anti-apoptotic proteins [e.g., BCL2 and BCL2-like 1 (BCL2L1/BCLXL)]. Members of the anti-apoptotic BCL2 protein family are overexpressed in many malignant tumors and become targets for tumor treatment (Adams and Cory, 2007). In addition to regulating mitochondrial apoptosis by controlling mitochondrial outer membrane permeabilization, the OMM protein BCL2 interacting protein 3-like (BNIP3L/NIX, a pro-apoptotic BCL2 family member) is involved in mediating mitophagy (Schweers et al., 2007; Sandoval et al., 2008). Unlike binding BCL2 during apoptosis (Zhang and Ney, 2009), BNIP3L directly binds to MAP1LC3 or GABARAP during mitophagy (Schwarten et al., 2009). Moreover, BNIP3L-mediated mitophagy may not be associated with the ubiquitination of BNIP3L, indicating that it is an Ub-independent receptor for mitophagy (Ney, 2015).

Functionally, the activation of BNIP3L-dependent mitophagy is essential for the programmed mitochondrial elimination during erythroid maturation, and BNIP3L-depleted mice show anemia (Schweers et al., 2007; Sandoval et al., 2008). Furthermore, BNIP3L instead of PRKN is responsible for mitophagy induction in HeLa cells (a cell line derived from patients with cervical cancer) (Ding et al., 2010). In addition, transcriptional factor hypoxia-inducible factor 1 subunit alpha (HIF1A/HIF1α)-mediated BNIP3L upregulation is required for hypoxia-induced mitophagy (Sowter et al., 2001), indicating a potential role of BNIP3L-mediated mitophagy in hypoxic tumor microenvironments (TMEs). Structurally, BNIP3L positioning on OMM requires the transmembrane domain, and BNIP3L dimerization is responsible for MAP1LC3 recruitment (Marinkovic et al., 2020). BNIP3L further binds to MAP1LC3 at the amino terminus of BNIP3L through the LIR motif (Schwarten et al., 2009). These structural studies provide information for the development of BNIP3L-targeted drugs.

#### BCL2 Interacting Protein 3

BCL2 interacting protein 3 (BNIP3) is a BH3-only protein and acts as a pro-apoptotic BCL2 family member (Vande Velde et al., 2000). It interacts with the anti-apoptotic BCL2, thereby overcoming the inhibitory effect of BCL2 on apoptosis (Zhang and Ney, 2009). BNIP3 at the OMM regulates the opening of the pores in the mitochondrial double membrane to mediate the transport of lysosomal proteins from the cytoplasm to the mitochondrial matrix, thereby leading to the degradation of damaged proteins in the mitochondria in response to oxidative damage (Zhang and Ney, 2009). BNIP3 also has a LIR domain through which it interacts with MAP1LC3 and mediates mitochondrial degradation through mitophagy (Novak et al., 2010; Park et al., 2013). Like its homolog BNIP3L, BNIP3 forms a dimer during mitophagy and its expression is regulated by HIF1A during hypoxia (Sowter et al., 2001; O’Sullivan et al., 2015). BNIP3 is also highly expressed in the hypoxic environment of solid tumors. Although both BNIP3 and BNIP3L mediate hypoxia-induced mitophagy (Sowter et al., 2001), the functional complementarity and differences of these two proteins in cancer-related mitophagy remain largely unclear. In addition, BNIP3 may affect the fission or fusion of mitochondria by binding to OPA1 mitochondrial dynamin-like GTPase (OPA1) or dynamin 1-like (DNM1L/DRP1), thereby promoting mitophagy (Lee et al., 2011). These findings highlight the role of mitochondrial dynamics in regulating mitophagy.

#### FUN14 Domain Containing 1

In addition to BNIP3L and BNIP3, FUN14 domain containing 1 (FUNDC1) was also found to be expressed in OMM as an autophagy receptor for mitophagy during hypoxia (Liu et al., 2012). The activity of FUNDC1 in hypoxia-induced mitophagy is regulated by phosphorylation and dephosphorylation events. Unc-51–like autophagy-activating kinase 1 (ULK1/ATG1) is the only kinase of the ATG family and a component of the ULK1-ATG13 RB1-inducible coiled-coil 1 (RB1CC1) complex, which initiates the formation of autophagosomes in mammalian cells (Hosokawa et al., 2009; Jung et al., 2009). ULK1 has many phosphorylation sites, and these phosphorylation sites have different functions in regulating autophagy (Xie et al., 2015). Phosphorylation of ULK1 at serine17 mediates ULK1 translocation to mitochondria and subsequently binds to FUNDC1 during hypoxia (Wu et al., 2014). In contrast, the dephosphorylation of FUNDC1 by PGAM family member 5, mitochondrial serine/threonine protein phosphatase (PGAM5) under hypoxia increases its binding to MAP1LC3 through LIR, and recruits the isolation membrane that binds to MAP1LC3, further forming autophagosomes to engulf damaged mitochondria in mammalian cells (Liu et al., 2012; Chen et al., 2014). Certain proteins [e.g., mitochondrial E3 ligase membrane-associated ring CH-type finger 5 (MARCHF5/MARCH5) and cytosolic molecular chaperone heat shock protein family A (hsp70) member 8 (HSPA8/HSC70)] bind to FUNDC1, which further regulate the protein stability of FUNDC1 to fine-tune mitophagy during hypoxia (Chen et al., 2017; Li et al., 2019b). Interestingly, the PRKN-mediated ubiquitination may promote the transport of MARCHF5 from mitochondria to peroxisomes, resulting in a decrease in mitophagy (Koyano et al., 2019). FUNDC1 also acts as a platform for regulating mitochondrial dynamics (e.g., fission and fusion) and mitophagy by interacting with DNM1L and OPA1 (Chen et al., 2016). In particular, the dissociation of FUNDC1 from DNML1 to form a complex with OPA1 inhibits mitochondrial fission and mitophagy (Chen et al., 2016). These findings further support the idea that mitochondrial dynamics and quality control are inseparably intertwined.

#### The 4-Nitrophenylphosphatase Domain and Non-neuronal SNAP25-Like Protein Homolog 1

Both 4-nitrophenylphosphatase domain and non-neuronal SNAP25-like protein homolog 1 (NIPSNAP1) and NIPSNAP2 play a major role in vesicular transport (Seroussi et al., 1998). Under normal conditions, they are located in the IMM and act as modulators of calcium channels (Brittain et al., 2012). However, they also localize to the OMM during mitochondrial depolarization to recruit autophagy receptors, MAP1LC3 homologs, and other proteins, and effectively serve as an “eat me” signal for triggering PRKN-dependent mitophagy (Princely Abudu et al., 2019). For example, the recruitment of autophagy receptors, such as calcium binding and coiled-coil domain 2 (CALCOCO2/NDP52), SQSTM1, NBR1 autophagy cargo receptor (NBR1), tax1-binding protein 1 (TAX1BP1), and WD repeat and FYVE domain containing 3 (WDFY3/ALFY), to depolarized mitochondria is mediated by NIPSNAP1 and NIPSNAP2 during mitophagy (Princely Abudu et al., 2019). Accordingly, NIPSNAP1 and NIPSNAP2, which require OMM localization, interact with MAP1LC3 or GABARAP as preferred interaction partners (Princely Abudu et al., 2019). Although zebrafish lacking Nipsnap1 show a decrease in mitochondria in the brain, which is coupled with the production of ROS, the loss of dopaminergic neurons, and a strong decrease in movement (Princely Abudu et al., 2019), the impact of NIPSNAP1 or NIPSNAP2-mediated mitophagy in neurodegenerative disease in mice or humans remains unknown.

#### BCL2-Like 13

BCL2-like 13 (BCL2L13/Bcl-rambo) is an OMM protein, a member of the pro-apoptotic BCL2 family with four conserved BH domains (Murakawa et al., 2015). The overexpression of BCL2L13 induces caspase-3–dependent apoptosis, which can be blocked by co-expression of inhibitor of apoptotic proteins (IAPs) (Kataoka et al., 2001). However, unlike other BCL2 members, BCL2L13 does not require the BH domains to induce apoptosis, but instead relies on mitochondrial localization by the transmembrane domain (Kataoka et al., 2001). In addition to promoting apoptosis, BCL2L13 also acts as a homolog of Atg32 in mammalian cells, mediating mitochondrial fragmentation and subsequent mitophagy (Murakawa et al., 2015). The OMM protein Atg32 is an autophagy receptor for mitophagy in yeast, and interacts with Atg8 and Atg11 (Liu et al., 2012). BCL2L13 interacts with MAP1LC3 through a conserved LIR sequence, leading to autophagosome engulfment of damaged mitochondria (Otsu et al., 2015). BCL2L13-mediated mitochondria also require fission mechanisms to drive mitochondrial fragmentation (Murakawa et al., 2015). The BCL2L13 gene is involved in a wide range of cancers, but whether BCL2L13-mediated mitophagy affects tumor development is still poorly understood.

#### Prohibitin 2

Prohibitin 2 (PHB2) is a conserved protein found in the mitochondria and the nucleus of eukaryotic cells, and plays a role in development, lifespan regulation, and various cellular processes (including mitochondrial dynamics) (Wei et al., 2017; Zhou et al., 2018). Notably, PHB2 was initially identified as a specific repressor of estrogen receptor in the nucleus by competitively inhibiting the binding between nuclear receptor coactivator 1 (NCOA1/SRC-1) and estrogen receptors (Montano et al., 1999; Kasashima et al., 2006). PHB2 can combine with PHB1 to form a large ring complex on the mitochondrial membrane and act as a molecular chaperone to stabilize mitochondrial proteins, thereby supporting mitochondrial morphogenesis and preventing cell death (Tatsuta et al., 2005). Moreover, mitochondrial PHB2 acts as an autophagy receptor for the clearance of damaged mitochondria in mammalian cells and *C. elegans* (Wei et al., 2017). In many cases, the IMM protein requires the rupture of the OMM to recruit the mitophagy molecular machinery (including mitophagy receptors and MAP1LC3) (Wei et al., 2017). However, in some cases, this dynamic positional change of the IMM protein is not necessary for mitophagy. Alternatively, PHB2 may act as a direct autophagy receptor in the IMM and binds to MAP1LC3 through the classical LIR motif, thereby degrading mitochondria (Wei et al., 2017). However, PHB2 promotes PINK1-PRKN–mediated mitophagy in a MAP1LC3-independent manner via the presenilin-associated rhomboid-like (PARL)-PGAM5 axis (Yan et al., 2020). Thus, both OMM receptors and IMM receptors participate in mitophagy-mediated mitochondrial removal.

#### FKBP Prolyl Isomerase 8

FKBP prolyl isomerase 8 (FKBP8/FKBP38) is a member of the immunophilin family, which has a conserved peptidyl prolyl *cis*/*trans*-isomerase domain. FKBP8 not only plays a role in immune regulation, but also participates in protein quality control (e.g., protein folding and trafficking) (Okamoto et al., 2006; Janssens et al., 2014; Xu et al., 2019). When combined with calmodulin and calcium, FKBP8 becomes active (Edlich et al., 2005). FKBP8 is anchored by the transmembrane domain and is mainly distributed in mitochondria (Shirane-Kitsuji and Nakayama, 2014). Mitochondrial FKBP8 acts as a molecular chaperone of BCL2 or heat shock proteins to inhibit apoptosis (Chen et al., 2008; Misaka et al., 2018). In addition to its anti-apoptotic function in response to various mitochondrial stresses, FKBP8 is also an autophagy receptor for damaged mitochondria (Bhujabal et al., 2017). FKBP8 has a typical LIR motif, and can strongly recruit MAP1LC3 to damaged mitochondria in HeLa cells during mitophagy (Bhujabal et al., 2017). Consequently, the overexpression of FKBP8 promotes mitochondrial fission, leading to mitophagy (Bhujabal et al., 2017). Unlike other autophagy receptors that usually degrade with cargo, FKBP8 escapes autophagosome degradation during mitophagy and instead relocates to the endoplasmic reticulum to bind BCL2 (Bhujabal et al., 2017). Thus, FKBP8 partially protects mitochondria from damage through mitophagy activation.

#### Mitochondrial Membrane Lipids

Cardiolipin is a diphosphatidylglycerol lipid, first found in animal hearts. It is an important component of the IMM, accounting for 20% of its total lipid composition (Paradies et al., 2014). In addition to mitochondria, cardiolipin can also be found in the membranes of most bacteria (Carranza et al., 2017). Cardiolipin homeostasis plays a key role in regulating mitochondrial function, and is involved in metabolism, cell death, and mitochondrial quality control (Dudek, 2017). For example, cardiolipin is necessary for the enzymatic activity of the respiratory chain complex and acts as a proton trap for oxidative phosphorylation (Dudek, 2017). The distribution of cardiolipin on the OMM not only triggers apoptosis, but also induces mitophagy to clear damaged mitochondria by interacting with MAP1LC3 (Chu et al., 2013), indicating that cardiolipin is an important eat me signal that regulates cell death and survival after mitochondrial injury.

Other lipids that contribute to mitophagy come from ceramides, which are composed of sphingosine and fatty acids. For example, C_18_-ceramide synthesized by ceramide synthase 1 (CERS1) induces mitophagy and tumor suppression in head and neck squamous cell carcinoma cells (Sentelle et al., 2012) and acute myeloid leukemia cells (Dany et al., 2016) *in vitro* and *in vivo*. Mechanistically, ceramide can bind to MAP1LC3 on the mitochondrial membrane to trigger mitophagy after DNM1L-mediated mitochondrial fission (Dany and Ogretmen, 2015). These findings provide another strategy for removing damaged mitochondria through the phospholipid components of the mitochondrial membrane.

### Mitophagy in Cancer

The role of autophagy in tumor biology is complex, which depends not only on the type of tumor, but also on the stage and context of the tumor (Levy et al., 2017). In general, autophagy plays a role in blocking the initiation of tumorigenesis because it inhibits genome instability and inflammation. In contrast, in established tumors, cancer cells may use autophagy to meet their metabolic requirements and enhance the resistance to cell death, leading to increased growth and invasiveness. Similarly, dysfunctional mitophagy is a characteristic phenomenon of cancer. Most mitophagy receptors or regulators are involved in cancer; however, whether they act as tumor promoters or tumor suppressors seems to be highly dependent on tumor type and TME (Table 1), which is described below (Panigrahi et al., 2019; Praharaj et al., 2019; Vara-Perez et al., 2019; Ferro et al., 2020).

**TABLE 1:** Role of mitophagy regulators in tumorigenesis.

Mitophagy regulator	Tumor type	Function in cancer	Mechanisms	References
BNIP3	Breast tumor	Tumor suppressor	Inhibits glycolysis and angiogenesis	Chourasia et al., 2015
BNIP3	Pancreatic cancer	Tumor suppressor	Promotes hypoxia-induced cell death	Okami et al., 2004
BNIP3	Colorectal cancer	Tumor suppressor	Promotes hypoxia-induced cell death	Murai et al., 2005; Bacon et al., 2007
BNIP3	Gastric cancer	Tumor suppressor	Promotes hypoxia-induced cell death	Murai et al., 2005
BNIP3L	Pancreatic ductal adenocarcinoma	Tumor promoter	Increases glucose metabolism and antioxidant capacity	Humpton et al., 2019
Ceramide	Head and neck squamous cell carcinoma, acute myeloid leukemia cells	Tumor suppressor	Promotes cell death	Sentelle et al., 2012; Dany et al., 2016
FUNDC1	Hepatocellular carcinoma	Tumor suppressor	Inhibits inflammation	Li et al., 2019a
FUNDC1	Laryngeal cancer	Tumor promoter	Promotes cell proliferation and survival	Hui et al., 2019
FUNDC1	Cervical cancer	Tumor suppressor	Promotes apoptosis	Hou et al., 2017
PHB2	Cervical/non-small cell lung/colorectal cancer cells	Tumor suppressor	Promotes activation of PINK1-PRKN pathway	Yan et al., 2020
PHB2	Non-small cell lung carcinoma	Tumor promoter	Promotes cell proliferation and migration	Zhang et al., 2020
PINK1	Pancreatic ductal adenocarcinoma	Tumor suppressor	Inhibits inflammation and antitumor immunity	Li et al., 2018
PRKN	Pancreatic ductal adenocarcinoma	Tumor suppressor	Inhibits inflammation and antitumor immunity	Li et al., 2018; Yin et al., 2018
PRKN	Colon cancer	Tumor suppressor	Inhibits cell proliferation	Poulogiannis et al., 2010

#### Mitophagy Inhibits Tumorigenesis

The PINK1-PRKN pathway is considered to be the main pathway of mitophagy in cancer cells (Bernardini et al., 2017). A loss of PINK1 or PRKN function impairs mitochondrial quality control, which further leads to the accumulation of ROS, thereby affecting cell function. The mutation or depletion of PINK1 or PRKN is often detected in a variety of tumors, such as lung cancer, glioma, and colon cancer (Bernardini et al., 2017). For example, the PRKN gene and human colorectal cancer are obviously associated with adenomatous polyps, and the expression of PRKN can inhibit the proliferation of colon cancer cells (Poulogiannis et al., 2010). The hybridization of PRKN knockout mice with colorectal adenomatous polyposis mice significantly accelerates the development of intestinal adenomas in newborn mice, and the diversity of polyps is also significantly increased, indicating that PRKN is a tumor suppressor gene in colon cancer (Poulogiannis et al., 2010). In addition, in a KRAS-driven tumor model, the depletion of PINK1 or PRKN promotes pancreatic tumorigenesis in mice (Li et al., 2018). Mechanistically, PINK1- and PRKN-mediated autophagy degradation of mitochondrial iron importers [e.g., solute carrier family 25 member 37 (SLC25A37) and solute carrier family 25 member 28 (SLC25A28)] suppresses pancreatic tumors by attenuating mitochondrial iron accumulation, inflammasome activation, high-mobility group box 1 (HMGB1) release, and subsequent immune checkpoint expression (Li et al., 2018). Therefore, the pharmacological or genetic inhibition of mitochondrial iron-dependent signaling prolongs the survival of animals and reverses the phenotype of mitophagy deficient-mediated pancreatic tumors *in vivo* (Li et al., 2018). These findings suggest that PINK1-PRKN pathway-mediated mitophagy links iron metabolism to tumor immunity during tumor formation (Kang et al., 2019). Unlike extracellular HMGB1 that promotes tumor growth, intracellular HMGB1 can regulate autophagy and mitophagy to inhibit the development of pancreatic cancer (Tang et al., 2010, 2011; Kang et al., 2017; Kang and Tang, 2018).

As discussed above, the activation of HIF1A increases the expression of BNIP3 and subsequent mitophagy. In turn, the expression of BNIP3 may affect HIF1A stability. This HIF1A-BNIP3–mediated mitophagy pathway is also implicated in controlling tumorgenesis in some cancers, such as triple-negative breast cancer (TNBC) (Chourasia et al., 2015). In fact, during the metastasis of TNBC, HIF1A-dependent BNIP3 expression is often suppressed or absent (Chourasia et al., 2015). The combination of BNIP3 deletion and high HIF1A expression predicts poor metastasis-free survival for TNBC (Chourasia et al., 2015). The increased aggressiveness of breast tumors in BNIP3-depleted mice is related to a decrease in mitophagy and the increased stability of HIF1A, indicating that BNIP3 can inhibit HIF1A and mitochondrial dysfunction (Chourasia et al., 2015). In addition, BNIP3 has a tumor suppressor effect in pancreatic cancer (Okami et al., 2004), colorectal cancer (Murai et al., 2005; Bacon et al., 2007), and gastric cancer (Murai et al., 2005), which is related to hypermethylation of the BNIP3 promoter. Whether the epigenetic silencing of BNIP3 can help reduce mitophagy and subsequent tumorigenesis remains unanswered.

FUNDC1 is another player in hypoxia-mediated mitophagy through its dephosphorylation (Liu et al., 2012). In cervical cancer, the expression of FUNDC1 was higher in tumors than in adjacent normal tissues (Hou et al., 2017). This high FUNDC1 expression is negatively correlated with tumor progression and patient prognosis, indicating a potential role of FUNDC1 in the suppression of tumor growth of cervical cancer (Hou et al., 2017). In addition, FUNDC1-mediated mitophagy protects laryngeal cancer cells against oxidative stress (Hui et al., 2019), which correlates with tumorigenic potential. Conditional knockout of FUNDC1 in the liver also initiates liver cancer by activating inflammation (Li et al., 2019a).

#### Mitophagy Promotes Tumor Progression

In some cases, the activation of a specific mitophagy pathway may promote tumor growth and development. Although both BNIP3 and BNIP3L are similar modulators of mitophagy and apoptosis, BNIP3L, unlike BNIP3 which inhibits tumorgenesis, plays an opposite role in promoting tumorigenesis. For example, the loss of BNIP3L in the KPC (*LSL-Kras^*G*12D^; Tp53^*R*172H^; Pdx1-Cre*) model of pancreatic ductal adenocarcinoma (PDAC) delays tumor occurrence, which is associated with reduced mitophagy and attenuated progression from the pancreatic intraepithelial neoplasia stage to PDAC (Humpton et al., 2019). These findings raise an unsolved question about the role of BNIP3L-dependent mitophagy in mutated KRAS and mutated TP53-driven tumorigenesis. One possibility is that different types of mitophagy may produce different TMEs, which further affects inflammation response and tumor immunity. It is also a challenge to distinguish the mitophagy-dependent and -independent role of BNIP3L in tumor biology.

#### Mitophagy and Tumor Therapies

The main reason for treatment failure in cancer is the resistance of cancer cells to drugs, which leads to tumor recurrence and metastasis. Dysfunctional autophagy and mitophagy lead to drug resistance through multiple mechanisms, including inhibiting cell death, especially apoptosis (Levy et al., 2017). Cancer stem cells (CSCs) are self-renewing cell types that contribute to tumor onset, expansion, drug resistance, recurrence, and metastasis after treatment (Reya et al., 2001; Ward and Dirks, 2007). Mitochondria are an important source of ROS within most cells, including cancer cells. Elevated ROS production is a powerful inducer of apoptosis during chemotherapy. Mitophagy-mediated mitochondria degradation limits the production of ROS, thereby exerting a cytoprotective effect during chemotherapy and helping CSCs resist apoptosis (Ianniciello et al., 2018; Levy et al., 2020). Reversing mitophagy-mediated protective mechanisms may be one of the ways to reverse CSC chemotherapy resistance (Wang et al., 2020). For example, leukemia stem cells (LSCs) are resistant to traditional chemotherapy drugs because LSCs can attain a lower rate of energy metabolism and ROS production through fission-dependent mitophagy (Pei et al., 2018). LSCs increase the expression of fission, mitochondrial 1 (FIS1) through the adenosine 5′-monophosphate-activated protein kinase (AMPK) pathway (Pei et al., 2018). Blocking FIS1 gene expression blocks the mitophagy pathway by inhibiting glycogen synthase kinase 3 (GSK3) activity (Pei et al., 2018). The use of GSK3 inhibitors to target the AMPK-FIS1-GSK3-mediated mitophagy pathway may become a radical cure for acute myeloid leukemia (Pei et al., 2018). Doxorubicin (brand name: adriamycin) is used to treat different types of cancers. The inhibition of mitophagy enhances the anticancer activity of doxorubicin in colorectal cancer cells (Yan et al., 2017). Higher mitophagic levels are also found in CSCs in cisplatin-resistant oral squamous cell carcinoma and oxaliplatin-resistant human colorectal cancer (Naik et al., 2018; Takeda et al., 2019), supporting a widely pro-survival role of mitophagy in various chemo-resistant cancer cells.

In radiotherapy, increased mitophagy can also promote survival, which is mediated by the PINK1-PRKN pathway. Therefore, the inhibition of PINK1-PRKN–mediated mitophagy restores the radiosensitivity of tumor cells (Zheng et al., 2015). Temozolomide-perillyl alcohol (TMZ-POH) conjugate induces lysosomal dysfunction and subsequent impaired mitochondrial flux in non-small cell lung cancer cells and makes them sensitive to radiation, thereby showing TMZ-POH as a potential radiosensitizer (Chang et al., 2018). Ionizing radiation can trigger a series of cellular DNA damage responses, and the dynamic interaction between these responses and mitophagy remains to be revealed.

Compared with chemotherapy and radiotherapy, immunotherapy (e.g., using cytokines, antibodies, or immune checkpoint inhibitors) has shown emerging and great potential in inhibiting tumor growth. Accordingly, more research has focused on the dual roles of mitophagy in immunotherapy. On the one hand, inhibition of the mitophagic axis enhances tumor necrosis factor-based immunotherapy to control the survival and progression of cervical and gastric cancer cells (Yan et al., 2018; Zhao et al., 2019). On the other hand, enhanced mitophagy may induce immunogenic cell death, thereby inhibiting tumor growth through the activation of cytotoxic T lymphocytes in liver cancer cells (Yu et al., 2020). These findings further support that mitophagy may be an effective target for modified tumor immunotherapy.

## Conclusion and Perspectives

Mitochondria are multifunctional organelles that play an important role in cancer through the synthesis of macromolecules, energy production, and cell death regulation. Understanding the regulation of mitochondrial morphology, function, biogenesis, fission and fusion dynamics, and degradation is important for the development of new anticancer strategies. Dysfunctional mitophagy is a feature of the TME in many cancers and plays multiple roles in regulating tumor metabolism. On the one hand, mitophagy prevents the accumulation of damaged mitochondria, thereby maintaining energy production for tumor growth. On the other hand, mitophagy may suppress tumors by limiting the production of ROS, which is a well-known factor in causing gene mutation and chromosomal instability. Therefore, it is not surprising that mitophagy is a regulator of tumor biology, acting as either a suppressor or a facilitator of tumorigenesis.

The identification of various mitophagy-related autophagic receptors (including mitochondrial OMM, IMM, or lipid components) has accelerated our knowledge of the complexity of mitophagy in tumor biology. Therefore, understanding the molecular mechanism and function of mitophagy during different types of mitochondrial stress and damage may be critical for developing the next generation of cancer treatment methods. It is also important to develop convenient and reliable methods or biomarkers to assess the activity of mitophagy in humans. In addition, distinguishing the function of mitophagy between normal cells and cancer cells may be important for improving the targeting of tumor therapy and reducing its toxicity.

## Author Contributions

YX and DT conceived of the topic for this review. All authors listed have made a substantial, direct, and intellectual contribution to the work, and approved it for publication.

## Conflict of Interest

The authors declare that the research was conducted in the absence of any commercial or financial relationships that could be construed as a potential conflict of interest.
